# Endoscopic Combined Intrarenal Surgery for a Large Stone in a Rare Fused Supernumerary Kidney

**DOI:** 10.7759/cureus.76438

**Published:** 2024-12-26

**Authors:** Francisco Gomez-Regalado, Said Castro-Zazueta, Alejandro Figueroa-Garcia, Carlos Francisco Tejeda-Andrade, Mario Eduardo Galland-Novelo

**Affiliations:** 1 Urology, Hospital Angeles del Carmen, Guadalajara, MEX; 2 Urology, Hospital General Dr. Agustín O'Horán, Mérida, MEX

**Keywords:** endoscopic combined intrarenal surgery (ecirs), endourology, fused kidney, percutaneous nephrolithotomy (pcnl), renal calculi (kidney stones), supernumerary kidney

## Abstract

A supernumerary kidney is a rare birth defect where an extra kidney is present. This extra kidney has its own separate outer covering, blood supply, and collection system. Normally, percutaneous nephrolithotomy (PCNL) is the treatment of choice for large kidney stones, but its ideal use for supernumerary kidneys is unknown. This report aims to show the success of a surgical technique called endoscopic combined intrarenal surgery (ECIRS) for removing a big stone from the fused supernumerary kidney of a patient.

A 48-year-old man experienced occasional mild chronic pain in his left flank and occasionally hematuria. An imaging scan showed a stone (2.5 cm, 924 Hounsfield units) in a fused supernumerary kidney on the left side. The stone was successfully removed with a single procedure of PCNL that consisted of PCNL and a retrograde ureteroscopy approach at the same surgical time.

ECIRS management of urinary tract calculi can be a consideration for patients presenting with fused supernumerary kidney and large stone, offering a reasonable success rate.

## Introduction

A rare congenital anomaly, a supernumerary kidney, is characterized by one or more extra kidneys present and is still rarer when they are fused together [1].

Renal stones over 2 cm are effectively treated with percutaneous nephrolithotomy (PCNL), with success rates ranging from 25% to 90% for simpler cases. The renal anatomy significantly impacts the likelihood of successful stone removal. Adequate pre-surgical planning is crucial to achieve the best results, particularly given the complexities that anatomical abnormalities and large stones present for endourology [2].

This clinical case aims to showcase the effectiveness of endoscopic combined intrarenal surgery (ECIRS) in a patient with a fused supernumerary kidney who had a large stone [1,3].

## Case presentation

A 48-year-old male presented to the emergency department who had mild recurring back pain on the left side and occasionally hematuria. The patient was otherwise healthy, with no significant medical history. Physical examination revealed no significant findings. A urine test confirmed hematuria, with 48 erythrocytes per high-power field.

**Figure 1:**
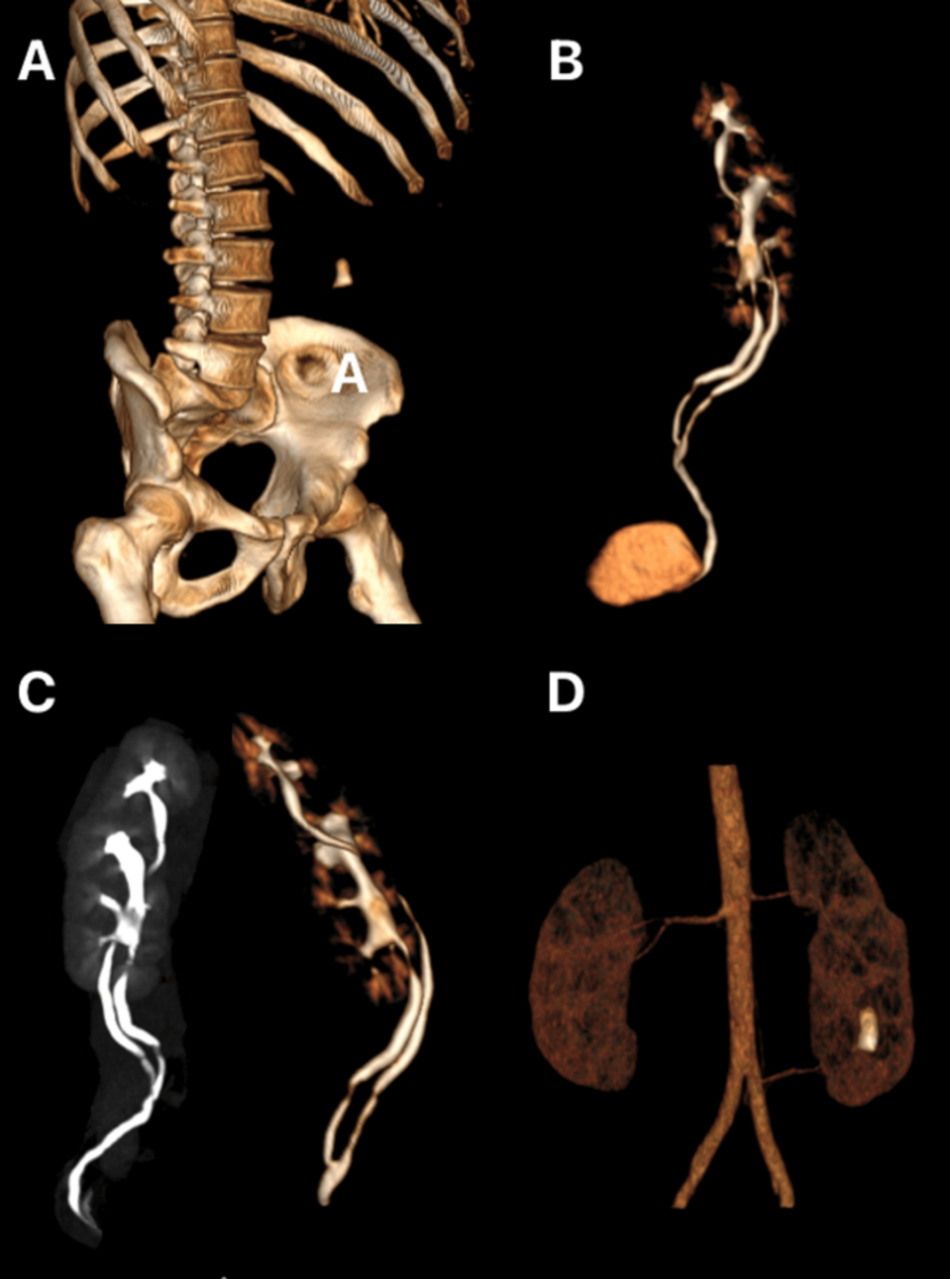
Angiotomography CT (A) 3D reconstruction showing just a large calculus. (B) 3D reconstruction of the urinary system's collecting system featuring the two separate renal pelvises and two ureters that unite (incomplete). (C) 3D reconstruction of the left supernumerary fused kidney. (D) Angiotomography CT: angiography reconstruction showing the vascular supply of the three kidneys (right, left, and ipsilateral supernumerary) with one artery each. The supernumerary (superior) kidney artery is identified as a direct branch of the aorta, and the native (inferior) kidney artery is a branch of the left common iliac artery.

An angiotomography showed a 2.5 cm kidney stone (924 Hounsfield units) in a joined supernumerary kidney on the left, having two separate renal pelvis and two ureters that unite. The ureter of the supernumerary (upper) kidney is seen laterally, and the ureter of the native left kidney is identified medially. These join at the level of the third portion of the ureter of the native left kidney (Figure 1). The urine culture was negative.

**Figure 2:**
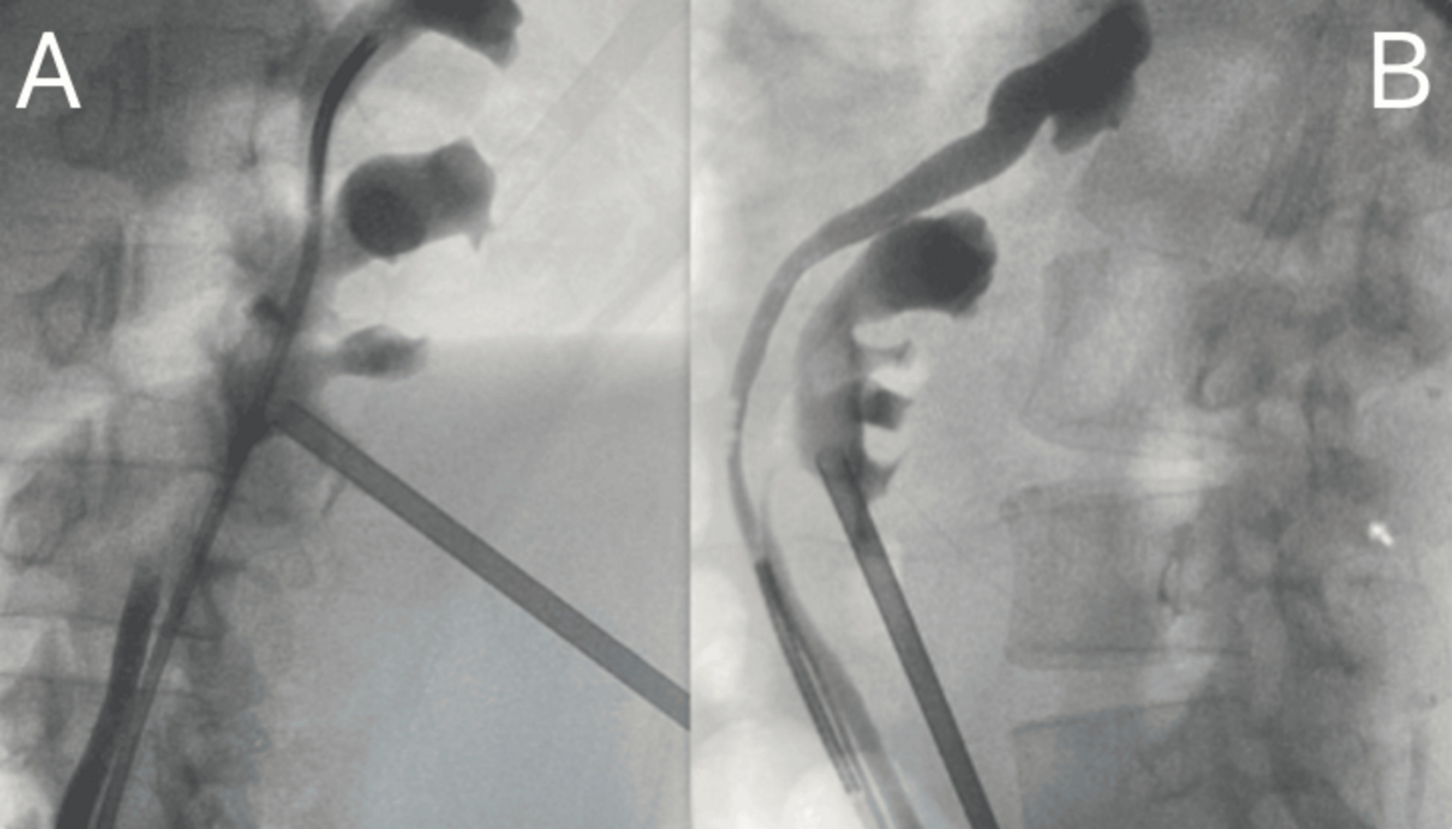
Fluoroscopic perioperative images Fluoroscopic perioperative images, with percutaneous tract retrograde flex ureteroscopy to get renal access (endoscopic combined intrarenal surgery (ECIRS)). (B) During the final stages of the surgery, a control retrograde pyelography picture was obtained.

The procedure was done while the patient was in a Galdakao-modified supine position. A flexible ureteroscopy was performed to examine and guide puncture to the kidney area (Figure 2).

Renal access was achieved using the simplified 0-90 fluoroscopic puncture technique. A single percutaneous puncture was made through the lower pole calyx of the native kidney. The tract was dilated using the "one-shot" dilation method with a 16F sheath. The Karl Storz® minimally invasive PCNL (MIP)-M nephroscope (Karl Storz SE & Co. KG, Tuttlingen, Germany) was used for the procedure. Lithotripsy was performed with a 100 W holmium YAG laser (Boston Scientific, Marlborough, MA). An 11F/13F ureteral access sheath and the Flex-X2 scope (Karl Storz SE & Co. KG, Tuttlingen, Germany) were employed for retrograde access. The procedure lasted 56 minutes with a minimal fluoroscopy of 34 seconds.

**Figure 3:**
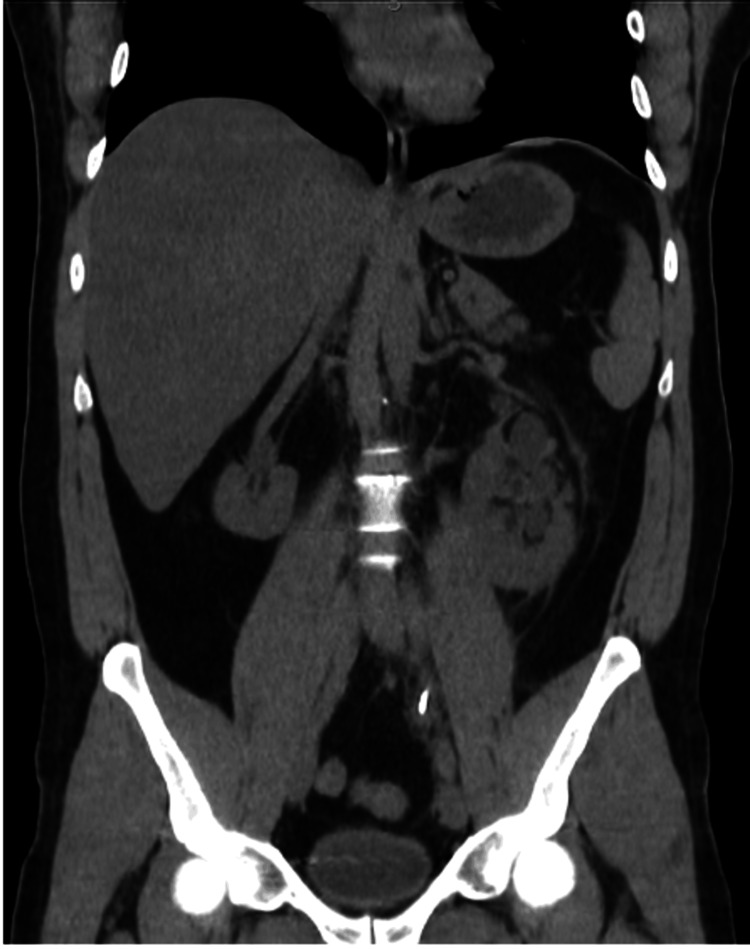
Postoperative nonenhanced CT Twenty-four hours after surgery, a non-enhanced CT scan showed a stone-free-rate status.

At the end of the procedure, the collecting system was thoroughly inspected with a nephroscope to check for any remaining stones, and none were found. The patient left the hospital the day following the procedure without complications. The double-J stent was removed one week later after a postoperative CT scan confirmed the complete absence of residual stone fragments (Figure 3).

## Discussion

A congenital double kidney on one side with a normal kidney on the other side can arise as a result of premature division of the ureteric bud in early development, then fusion of the lower pole of the kidney with the upper pole of the kidney below can occur with further development [4].

Congenital malformations of the urinary system account for approximately 20% to 30% of all prenatal malformations [5].

Compliant with European Association of Urology (EUA) stipulations, PCNL remains the paramount procedure for large and complex renal calculi, demonstrating its effectiveness to other treatment modalities despite the stone size, thereby establishing its status as the gold standard treatment [6].

Additionally, if a stone is lodged in the target calyx or its infundibulum, retrograde holmium laser lithotripsy can drill a passage through the calculus, allowing the ureteroscope to expose the target calyx, visualize the puncture site, or facilitate the descending guidewire and the free flow of irrigation.

Managing complex renal calculi presents substantial therapeutic challenges, characterized by elevated rates of complications and retreatment. The optimal therapeutic strategy for renal lithiasis in aberrant renal morphology currently lacks consensus [7]. A recent retrospective analysis involving 48 patients experiencing renal calculi concomitant with various renal anomalies (inclusive of renal fusion, ectopia, rotation, hypoplasia, and pelvicalyceal dysmorphia) subjected to PCNL demonstrated a striking stone-free rate (SFR) of 81% following a solitary treatment session, with calculi averaging 39 mm in diameter. This outcomes-based evidence supports PCNL as a good treatment option for large renal calculi in kidneys exhibiting deviant morphological features [8].

Division of the patient population into minimally invasive and conventional subgroups revealed a consensus in favor of ECIRS, with additional benefits observed in the minimally invasive subgroup, namely, shorter hospital stays and reduced postoperative fever [9,10].

A comparative analysis has demonstrated that ECIRS exhibits superior results when treating complex renal calculi, as evidenced by improved initial and final SFRs, reduced overall and severe complications, and decreased requirement for blood transfusions in comparison to PCNL [10].

With our patient, a comprehensive approach combining anterograde and retrograde techniques was successfully employed in the treatment of a substantial stone with a fused supernumerary kidney, yielding complete stone clearance in a solitary session.

Studies suggest that patients with large and big urolithiasis may derive considerable benefit from ECIRS. The integration of retrograde flexible ureteroscopy during PCNL serves a dual function, facilitating both diagnostic evaluation and therapeutic intervention [11].

ECIRS may be viewed as an updated, advanced, and good adaptation of traditional PCNL, offering flexibility and increased efficacy [11].

## Conclusions

We suggest considering ECIRS-like treatment for patients with a fused supernumerary kidney who have a large calculus, offering a reasonable success rate.

The anatomical variants of this patient represented a surgical challenge that was well treated, in our opinion, due to the favorable results obtained.

## References

[1] Berhe T, Hassen SM, Gebrehiwot FG (2021). Fused supernumerary kidney with single pelvis and ureter; presenting with stones: a case report and literature review. Res Rep Urol.

[2] Gómez-Regalado F, Manzo BO, Figueroa-Garcia A, Sanchez-Lopez H, Basulto-Martinez M, Cracco CM, Scoffone CM (2020). Efficacy of the endoscopic combined intrarenal surgery for the treatment of a staghorn calculus in crossed fused renal ectopia. J Endourol Case Rep.

[3] Scoffone CM, Cracco CM (2018). Invited review: the tale of ECIRS (Endoscopic Combined IntraRenal Surgery) in the Galdakao-modified supine Valdivia position. Urolithiasis.

[4] Hegazy A (2014). Clinical embryology for medical students and postgraduate doctors. https://www.researchgate.net/publication/263844854_Clinical_Embryology_for_medical_students_and_postgraduate_doctors.

[5] Arumugam S, Subbiah NK, Mariappan Senthiappan A (2020). Double ureter: incidence, types, and its applied significance—a cadaveric study. Cureus.

[6] Ruhayel Y, Tepeler A, Dabestani S (2017). Tract sizes in miniaturized percutaneous nephrolithotomy: a systematic review from the European Association of Urology Urolithiasis Guidelines Panel. Eur Urol.

[7] Serrate Aguilera RG, Urmeneta Sanromá JM, Banús Gassol JM, Regué Aldosa R, Rius Espina G, Prats López J (1991). New therapeutic alternative for complex renal lithiasis. Actas Urol Esp.

[8] Rana AM, Bhojwani JP (2009). Percutaneous nephrolithotomy in renal anomalies of fusion, ectopia, rotation, hypoplasia, and pelvicalyceal aberration: uniformity in heterogeneity. J Endourol.

[9] Sabler IM, Katafigiotis I, Gofrit ON, Duvdevani M (2018). Present indications and techniques of percutaneous nephrolithotomy: what the future holds?. Asian J Urol.

[10] Liu YH, Jhou HJ, Chou MH (2022). Endoscopic combined intrarenal surgery versus percutaneous nephrolithotomy for complex renal stones: a systematic review and meta-analysis. J Pers Med.

[11] Cracco CM, Scoffone CM (2020). Endoscopic combined intrarenal surgery (ECIRS) - tips and tricks to improve outcomes: a systematic review. Turk J Urol.

